# Magnesium-containing intramedullary nails promote fracture healing in type 2 diabetic animal model via recruiting regulatory T cells into the fracture callus

**DOI:** 10.1016/j.bioactmat.2026.07.014

**Published:** 2026-07-14

**Authors:** Shi'an Zhang, Bingyang Dai, Yuanming An, Zheyu Jin, Weiyang Liu, Naping Xiong, Hongwei Shao, Zhengming Shan, Lizhen Zheng, Xu Li, Yanbin Zhu, Jiankun Xu, Dick Ho Kiu Chow, Ronald Man Yeung Wong, Ling Qin, Wenxue Tong

**Affiliations:** a Musculoskeletal Research Laboratory of Department of Orthopaedics & Traumatology, Innovative Orthopaedic Biomaterial and Drug Translational Research Laboratory, Li Ka Shing Institute of Health Sciences, The Chinese University of Hong Kong, Hong Kong Special Administrative Region of China, China; b Department of Biomedical Engineering, Faculty of Engineering, The Hong Kong Polytechnic University, Hong Kong Special Administrative Region of China, China; c The Sir Yue-Kong Pao Cancer Centre, Prince of Wales Hospital, The Chinese University of Hong Kong, Hong Kong Special Administrative Region of China, China; d Centre for Regenerative Medicine and Health, Hong Kong Institute of Science and Innovation, Chinese Academy of Sciences, Hong Kong Special Administrative Region of China, China; e Centre for Musculoskeletal Degeneration and Regeneration, Department of Orthopaedics & Traumatology, Faculty of Medicine, The Chinese University of Hong Kong, Hong Kong Special Administrative Region of China, China; f Department of Orthopaedic Surgery, Hebei Medical University Third Hospital, Shijiazhuang, China

## Abstract

Magnesium-containing intramedullary nails (Mg-IMN) have been shown to promote fracture healing across various types of fractures, including osteoporotic and atypical femoral fractures. However, their role in the challenging chronic inflammatory type 2 diabetes (T2D) fracture healing remains unclear. In this study, we investigated the effect of Mg-IMN on fracture healing in a T2D mouse model and explored the involvement of regulatory T cells (Tregs) in this process. Our results demonstrated that Mg-IMN promotes fracture healing in Lepr^db/db^ T2D mice. At 5 days post-fracture, flow cytometry showed an increased number of Tregs in the fracture callus. Notably, depletion of Tregs via injection of PC61 neutralizing antibody abolished the promotive effect of Mg-IMN, indicating the critical role of Tregs in this process. Bulk RNA sequencing of the bone callus at 5 days post-fracture revealed significant enrichment of pathways related to chemokine signaling and CCR chemokine receptor interactions, suggesting a mechanism of Treg recruitment. Transwell migration assays *in vitro* preliminarily indicated the chemotactic effect of CCL19 and CCL21 in recruiting Tregs. Furthermore, Mg^2+^ treatment enhanced the mRNA expression levels of amphiregulin (*Areg*) and granulin (*Grn*) in Tregs, as indicated by qRT-PCR analysis. These findings may pave the way for new applications of Mg-IMN in fracture repair and provide unique insights into osteoimmunology during the complex process of T2D fracture healing.

## Highlights

• Mg-IMN accelerates T2D fracture healing by enhancing callus structural integrity and mechanical strength in Leprᵈᵇ/ᵈᵇ mice.
• Mg-IMN increases Treg infiltration in the callus of Lepr^db/db^ mice at 5 days post-fracture.
• Treg depletion impairs the effect of Mg-IMN on promoting T2D diabetic fracture healing.
• Tregs are recruited to the fracture site in response to the CCL19/CCL21-CCR7 axis after Mg-IMN implantation.

## Graphical abstract

**Figure:**
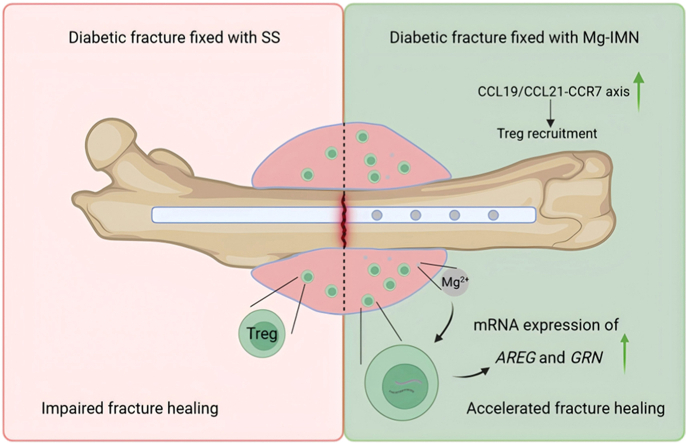

## 1 Introduction

The findings of the International Diabetes Federation (IDF) 11th edition confirm that diabetes is one of the fastest-growing global health emergencies [1]. Amongst the different kinds of types, type 2 diabetes (T2D) is the most prevalent, accounting for over 90% of diabetes cases worldwide [2]. T2D is a chronic metabolic disease characterized by persistent hyperglycemia, lack of insulin, and insulin resistance that induces long-term complications affecting multiple systems [2]. The impact of T2D on bone health has also been widely reported [3]. Extensive studies have demonstrated a significantly increased risk of fracture in individuals with T2D, particularly in the hip and non-vertebral bones, despite these patients often exhibiting normal or even elevated bone mineral density (BMD) [4,5]. However, in addition to the increased fracture risk, accumulating evidence indicates that bone fracture healing is also impaired in T2D patients, leading to delayed recovery and a higher likelihood of nonunion or malunion [6,7]. Several pathophysiological mechanisms have been identified that contribute to the impaired bone healing process [6]. Factors such as chronic low-grade inflammation, excessive accumulation of advanced glycation end-products (AGEs) and reactive oxygen species (ROS), persistent hyperglycemia, and dysregulated insulin signaling pathways exacerbate the chronic inflammation. Enhanced osteoclast activity, which suppresses osteoblast function, ultimately leads to impaired bone regeneration.

Magnesium (Mg) is an essential element for the human body, playing a crucial role in bone health and homeostasis [8], while Mg deficiency is reported to be associated with osteoporosis [9]. In recent years, Mg-based implants have attracted widespread attention in orthopaedics, attributed to their excellent biocompatibility, biodegradability, and bioactivity [10,11]. Based on our previous studies, Mg^2+^ derived from Mg-containing biodegradable orthopaedic implants enhances fracture healing by promoting neuronal production of calcitonin gene-related peptide (CGRP) [12]. Our novel Mg-containing intramedullary nail (Mg-IMN), which combines the mechanical support of a stainless steel nail (SS) and bioactive Mg, has demonstrated good effects in promoting fracture healing in preclinical osteoporotic fractures [13], atypical femoral fractures [14], and severe bone defects [15], as healing is often compromised in these situations [16,17]. Given that T2D is characterized by a chronic inflammatory microenvironment that severely impairs bone regeneration, in which the immunomodulatory and pro-healing properties of Mg are particularly advantageous, we aimed to evaluate the therapeutic potential of Mg-IMN specifically in the T2D fracture model.

Regulatory T cells (Tregs) represent a distinct subset of T cells that play a crucial role in modulating the immune system, maintaining immune tolerance to self-antigens, and preventing autoimmune diseases [18]. In addition to their well-established immunoregulatory functions, Tregs have been reported to facilitate tissue healing and regeneration in multiple organs, including skin, bone, skeletal muscle, and myocardium [19,20]. Recent studies emphasize the significant role of Tregs in fracture healing by modulating the osteogenic differentiation of various stem cell populations, including skeletal stem cells (SSCs) [21] and mesenchymal stem cells (MSCs) [22]. Clinical evidence also supports this effect, as T2D patients suffering delayed fracture healing exhibit reduced Treg and impaired immunoregulatory function [23,24]. Moreover, both the number and function of Tregs are diminished in T2D patients and mouse models, contributing to a chronic inflammation microenvironment [25,26]. Given this evidence, targeting Tregs to enhance T2D diabetic fracture healing presents a promising therapeutic approach. In this study, we hypothesize that Mg-IMN may promote fracture healing in T2D conditions, with Tregs playing an important role as mediators in this process. To explore this hypothesis, we investigated changes in Treg populations within the fracture callus with or without Mg-IMN implantation in T2D mice. We also explored the mechanisms underlying Treg recruitment and accelerated bone regeneration facilitated by Tregs, which contribute to Mg-IMN-enhanced fracture healing in T2D.

## 2 Materials and methods

### 2.1 Animals

Eight-week-old male Lepr^db/+^ and Lepr^db/db^ mice, a well-established T2D model with a leptin receptor inactivation mutation to induce continuous eating, were obtained from the Laboratory Animal Centre of the Chinese University of Hong Kong (CUHK). Previous studies showed that young adult Lepr^db/db^ mice already exhibited defective bone metabolism at experiment initiation, with reduced BMD, poor mechanical performance and delayed fracture healing [7]. All mice were maintained in a controlled environment with a 12 h light/dark cycle and access to a standard rodent diet and water. All experimental procedures were approved by the Animal Experiment Ethics Committee of CUHK (Ref. No.22-242-MIS-5-C and 23-174-MIS-5-C). Age-matched animals were randomly divided into groups for different treatments.

### 2.2 Fabrication of the magnesium-containing intramedullary nail (Mg-IMN)

23 G needles (hollow, outer diameter: 0.6 mm; inner diameter: 0.3 mm) were purchased from the Terumo company (Terumo, USA). These needles were sent to Ziyoujian Ltd. (Shenzhen, China) to have holes drilled in the cross-section along the vertical axis using electric sparks to facilitate Mg degradation and Mg^2+^ release *in vivo*, with a spacing of 1 mm between each hole. Subsequently, ultrapure (99.99%) Mg wire with a diameter of 0.3 mm and a length of 12 mm was inserted into the hollow needle to create the magnesium-containing intramedullary nail (Mg-IMN). The holes in the needle wall functioned as windows for the release of Mg^2+^. This hybrid system combines the mechanical support of stainless steel with the bioactive stimulation from the Mg wire *in vivo* with implantation over time.

### 2.3 Establishment of the unilaterally closed fracture model and implantation model in mice

Eight-week-old male Lepr^db/+^ and Lepr^db/db^ mice were used to establish the closed femoral fracture model and implantation model on the right femur, as previously described [27]. A 23-G stainless steel needle (SS) served as the control. Briefly, after anesthetizing the mice, an incision was made at the right knee, and the patella was dislocated to expose the distal femur. An SS or Mg-IMN was inserted through the femoral bone marrow cavity. For the fracture model, a transverse fracture was created in the medial axis of the right femur by allowing a 50 g weight to free-fall from 20 cm. The wound was sutured with 5-0 surgical sutures. For pain management, Temgesic was administered daily for three consecutive days postoperatively.

### 2.4 Intraperitoneal glucose tolerance test

Mice were fasted overnight for 16 h and then received an intraperitoneal (i.p.) injection of 20% glucose solution at a dose of 2 g/kg. Blood glucose concentrations were measured using Freestyle Lite blood glucose test strips and a meter, taking samples from the mouse tail tips before the injection and at 15, 30, 60, 90, and 120 min afterwards.

### 2.5 Micro-CT analysis

After collection of the fractured femora, the SS or Mg-IMN was removed according to the established protocol [12]. The femora were scanned by the μCT-40 imaging system (Scanco Medical, Brüttisellen, Switzerland), according to our established protocol [27]. The scan for the region of interest (ROI) was set to be 3 mm (200 slides) at the region of the femoral mid-shaft with a resolution of 15 μm per voxel. At week 2 after fracture, the reconstructed images between thresholds 158 and 211 represented the newly formed callus. At weeks 4 and 8 post-fracture, the reconstructed images between thresholds 211 and 1000 represented the newly formed mineralized callus and primary cortical bone. The quantitative analysis involved all 200 slides of the 2D images. 3D reconstruction of the mineralized tissue was performed with a low-pass Gaussian filter (Sigma = 0.8, Support = 1). Morphometric parameters included bone volume (BV), tissue volume (TV), the ratio of BV to TV (BV/TV), trabecular bone number (Tb.N), trabecular bone separation (Tb.Sp), bone mineral density (BMD), and tissue mineral density (TMD). For analysis of the cortical bone of the unfractured femora, the scan for the ROI was set to be 0.6 mm (40 slides) at the region of the femoral mid-shaft with a resolution of 15 μm per voxel. The quantitative analysis involved all 40 slides of the 2D images. 3D reconstruction of the mineralized tissue was performed with a low-pass Gaussian filter (Sigma = 0.8, Support = 1). The cortical thickness (Ct.Th) of cortical bone was measured using ImageJ. The scanning range for the Mg-IMN ROI was set to 12 mm, with a voxel resolution of 10 μm. The ROIs of Mg-IMNs were manually delineated on 2D slices of each scan to perform 3D reconstruction using a gray value threshold.

### 2.6 Histological analysis

After micro-CT analysis, the fractured femora were fixed in 4% paraformaldehyde (PFA) at 4 °C for 24 h and decalcified in a 12.5% ethylenediaminetetraacetic acid (EDTA) solution at room temperature for 14 days, followed by dehydration in a series of ethanols and xylene. The decalcified samples were then embedded in paraffin, and sections with a thickness of 5 μm were cut using a rotary microtome (RM2255, Leica, Germany) for H&E and Safranin O/Fast Green staining.

### 2.7 Biomechanical test

A mechanical testing machine (H25KS Hounsfield Test Equipment, Redhill, Surrey, UK) equipped with a 250 N load cell was used to perform the four-point bending biomechanical test, according to our established protocol [28]. The femora were positioned horizontally on the lower supporting bars at 8 mm apart, while the upper bars were at 4 mm apart, flanking the fracture callus. A compression load was applied at a compression speed of 5 mm/min until failure. Maximum load (N), stiffness (N/mm), and energy to failure (J) were calculated from the load-displacement curve using Vernier Graphical Analysis software.

### 2.8 Flow cytometry

Callus, blood, and spleen were freshly isolated from mice 5 days post-fracture according to the previous study [22]. After mechanical dissection, the callus was digested with 0.1% Collagenase A (Sigma, 10103578001) at 37 °C under 5% CO_2_ for 6 h, followed by filtration through a 70 μm cell strainer. Cells were collected and washed with phosphate-buffered saline (PBS), followed by immunostaining. The spleen was passed through a 70 μm cell strainer and washed with PBS. Red blood cells (RBC) in the blood and spleen were removed with RBC lysis buffer (Biolegend, 420302) at room temperature for 10 min. The cells were collected and washed with PBS, followed by immunostaining. For mouse tissues, we targeted the following antigens: CD45 (Biolegend, 103155, BV605), CD3 (Biolegend, 100274, PE-Cy5), CD4 (Biolegend, 100510, FITC), CD25 (Biolegend, 102034, BV421), and Foxp3 (Biolegend, 118904, PE). Samples were fixed using a 4% PFA or Foxp3/transcription factor fixation/permeabilization set (eBioscience, 00-5523-00) according to the manufacturer's instructions. Samples were acquired by FACSCelesta (BD Biosciences), and the data were analyzed using FlowJo software (v10.9.0).

### 2.9 Treg depletion

For Treg depletion, the neutralizing antibody anti-CD25 mAb PC61 (BioLegend, 102002) or control IgG (BioLegend, 401902) was administered to 8-week-old Lepr^db/db^ mice at 60 μg per mouse via i.p. injection. This treatment was given twice a week, starting one week before the fracture and continuing until two weeks after the fracture.

### 2.10 Bulk RNA sequencing

Callus from fractured Lepr^db/db^ + SS and Lepr^db/db^ + Mg groups was collected in TRIzol (15596026, Thermo Fisher Scientific) at 5 days post-fracture and immediately stored in liquid nitrogen. The callus samples were sent to Guangzhou Climb Biological Technology Company for library preparation and sequencing. Volcano maps of significantly differentially expressed genes (DEGs), a heatmap of DEGs, and Gene Ontology (GO) enrichment analysis were conducted. Kyoto Encyclopedia of Genes and Genomes (KEGG) pathway enrichment analysis was conducted. DEGs were defined as those with | log2 fold change | > 1 and p < 0.05. For each experimental group, a total of six mice were used. Calluses from two mice were pooled together to form one biological sample to ensure sufficient RNA yield and reduce individual variability.

### 2.11 T cell isolation

T cells were isolated from mice's spleens using a T cell isolation kit (19851, STEMCELL) according to the manufacturer's protocol. The isolated T cells were cultured in Roswell Park Memorial Institute 1640 medium (11875093, Thermo Fisher Scientific) supplemented with Treg Differentiation Supplement (10957, STEMCELL) and activated with Dynabeads mouse anti-CD3/CD28 beads (11452D, Thermo Fisher Scientific) for 120 h. After this period, the cells were collected for *in vitro* experiments.

### 2.12 Transwell migration assay

To assess the chemotactic response of Tregs to CCL19 and CCL21, a Transwell migration assay was performed using 12-well Transwell chambers with an 8 μm permeable membrane (Corning, USA). Briefly, recombinant mouse CCL19 (587802, Biolegend) and CCL21 (586402, Biolegend) were diluted in RPMI-1640 medium to a final concentration of 20 ng/mL and added to the lower chambers (500 μL/well). Purified Tregs (1 × 10^5^ cells in 250 μL RPMI-1640) were then placed in the upper chambers. After incubation at 37 °C with 5% CO_2_ for 3 h, the cells in the top and bottom chambers were collected and counted using flow cytometry.

### 2.13 *In vitro* cell culture

To evaluate the effects of degradation products, Tregs were cultured under various conditions following 120 h of *in vitro* differentiation. The control groups were maintained in Roswell Park Memorial Institute 1640 medium (RPMI 1640, 11875093, Thermo Fisher Scientific). For Mg^2+^ treatment, Tregs were treated with a gradient of Mg^2+^ concentrations in the control medium. To assess the influence of alkaline pH, Tregs were cultured in media adjusted to various pH values. For the high glucose (HG) group, cells were cultured in high-glucose DMEM (11965092, Thermo Fisher Scientific). In the hydrogen treatment group, Tregs were cultured in H2-rich medium. All cell cultures were incubated at 37 °C with 5% CO_2_ for 24 h.

### 2.14 Real-time quantitative PCR

Total RNA was isolated from Tregs using TRIzol™ reagent (15596026, Thermo Fisher Scientific). Complementary DNA (cDNA) was synthesized from the extracted RNA using the High-Capacity RNA-to-cDNA Kit (4387406, Thermo Fisher Scientific). Quantitative real-time PCR (qRT-PCR) was performed on a LightCycler® 480 system (Roche Applied Science, Indianapolis, IN, USA) with LightCycler® 480 SYBR Green I Master Mix (Roche), following the manufacturer's protocol. Gene expression levels were normalized to β-actin and analyzed using the 2−ΔΔCT method. The primer sequences used in this study are provided in Supplementary Table 1.

### 2.15 Statistical analysis

The sample size estimation was based on a power analysis of results from previous studies on bone fracture healing. Micro-CT analysis was used to estimate the statistical power of the current study. The primary endpoint was the degree of fracture healing at 8 weeks post-fracture, assessed by Micro-CT analysis, including BV/TV and BV. Secondary endpoints included biomechanical strength of the callus assessed by four-point bending mechanical test and flow cytometric analysis of Tregs in the callus. A statistical power of 0.80 with a significance level set at 0.05 (two-tailed) to detect the difference between Mg-IMN implantation and controls was chosen to calculate the sample size by G∗Power v3.1. Therefore, a sample size of 8 would provide sufficient statistical power to detect differences between control and treatment groups.

Mice were assigned to groups randomly. All numerical data are presented as mean ± SD. Statistical analyses were performed using the unpaired two-tailed Student's *t*-test for between-group comparisons and one-way or two-way ANOVA with Tukey's *post hoc* tests for multiple-group comparisons. A p-value of <0.05 was considered statistically significant. GraphPad Prism software (Version 9.5.0, San Diego, CA, USA) was used for all statistical analyses.

## 3 Results

### 3.1 Implantation of Mg-IMN does not change T2D conditions in Lepr^db/db^ mice

**Fig. 1:**
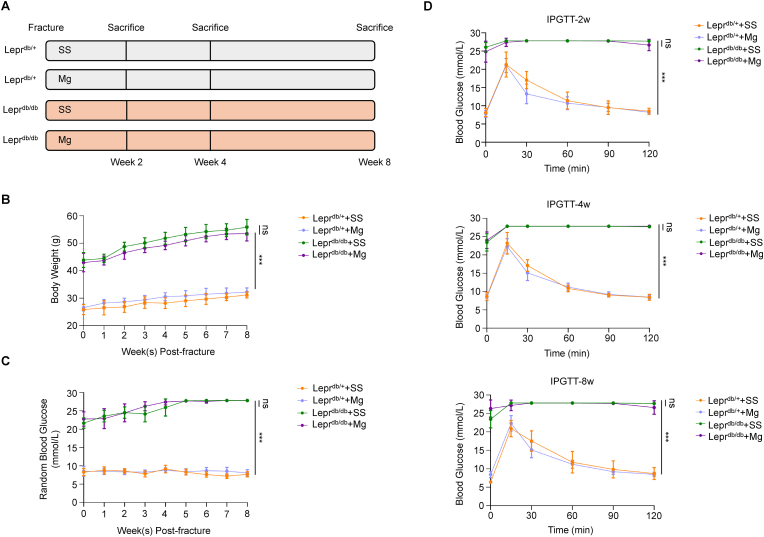
**Implantation of Mg-IMN does not rescue the T2D conditions in Lepr^db/db^ mice.** (A) Schematic diagram showing the experimental design and timeline of Lepr^db/+^ and Lepr^db/db^ mice with closed femoral fractures. (B) Comparison of the body weight between Lepr^db/+^+SS, Lepr^db/+^+Mg, Lepr^db/db^ + SS, and Lepr^db/db^ + Mg groups from 0 to 8 weeks post-fracture. ∗∗∗p < 0.001; ns, not significant, by two-way ANOVA with Tukey's *post-hoc* test. Data are presented as mean ± SD. n = 8 per group. (C) Comparison of the random blood glucose between Lepr^db/+^+SS, Lepr^db/+^+Mg, Lepr^db/db^ + SS, and Lepr^db/db^ + Mg groups from 0 to 8 weeks post-fracture. ∗∗∗p < 0.001; ns, not significant, by two-way ANOVA with Tukey's *post-hoc* test. Data are presented as mean ± SD. n = 8 per group. (D) IPGTT results of Lepr^db/+^+SS, Lepr^db/+^+Mg, Lepr^db/db^ + SS, and Lepr^db/db^ + Mg groups at 2-, 4-, and 8-weeks post-fracture. ∗∗∗p < 0.001; ns, not significant, by two-way ANOVA with Tukey's *post-hoc* test. Data are presented as mean ± SD. n = 8 per group.

To investigate the therapeutic potential of Mg-IMN on T2D diabetic fracture healing, we established closed transverse femoral fractures in 8-week-old Lepr^db/+^ and Lepr^db/db^ male mice and fixed them with stainless steel nails (SS) or Mg-IMNs (Fig. 1A). Given the documented involvement of magnesium in insulin signaling and diabetes pathogenesis [29], we systematically monitored systemic metabolic parameters throughout the 8-week postoperative period (Fig. 1B and C). The Lepr^db/db^ + Mg group exhibited similar characteristics of overweight and hyperglycemia as the Lepr^db/db^ + SS group compared to the Lepr^db/+^+Mg group, indicating that the Mg-IMN implantation did not significantly alter the T2D diabetic phenotype. To further evaluate metabolic function, we performed intraperitoneal glucose tolerance tests (IPGTT) at 2, 4, and 8 weeks after fracture (Fig. 1D). The results indicated that both the Lepr^db/db^ + SS and Lepr^db/db^ + Mg groups exhibited persistent glucose intolerance at all time points, suggesting the implantation of Mg-IMN did not have a significant rescue effect on glucose metabolism in T2D mice. These comprehensive metabolic analyses conclusively demonstrate that the therapeutic effect of Mg-IMN on diabetic fracture healing is mediated through local effects rather than systemic modulation of diabetic conditions. The *in vivo* degradation behavior of Mg-IMNs in the femora of T2D Lepr^db/db^ mice was evaluated by measuring volume change over an 8-week post-fracture period (Supplementary Fig. 1). The Mg-IMNs exhibited gradual and continuous volume loss after implantation. The volume decreased rapidly within the first 4 weeks, followed by a slower degradation rate from week 4 to week 8.

### 3.2 Mg-IMN promotes diabetic fracture healing in Lepr^db/db^ mice

**Fig. 2:**
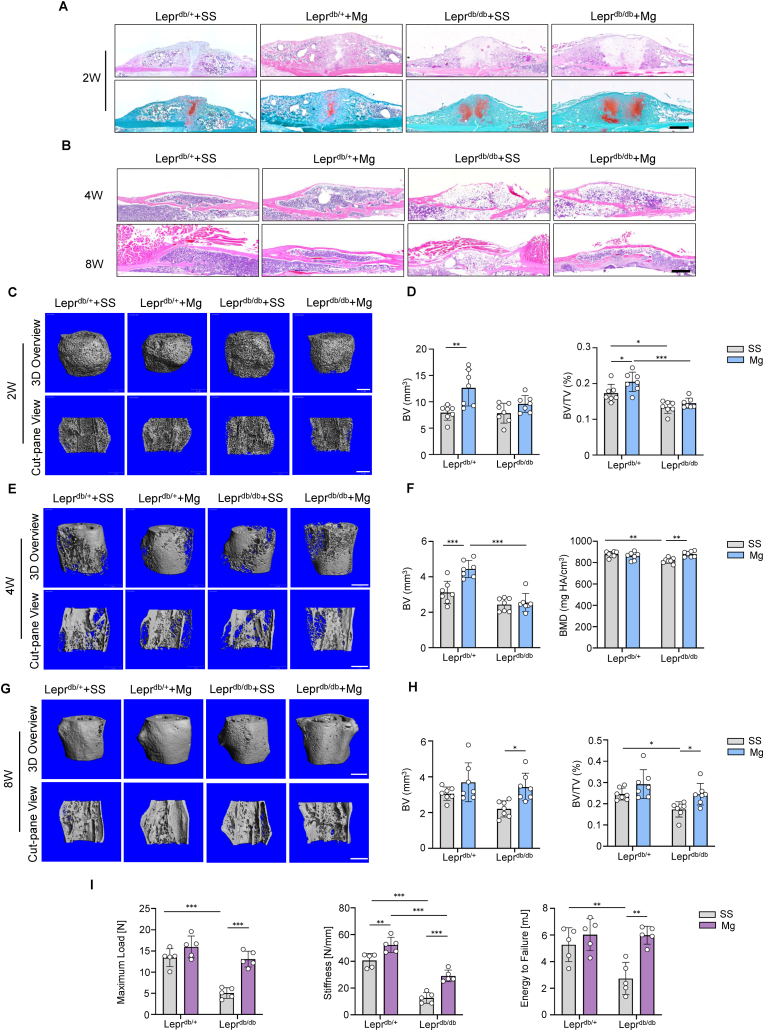
**Mg-IMN promotes impaired diabetic fracture healing in Lepr^db/db^ mice.** (A) Representative H&E and Safranin O/Fast Green staining of callus at 2 weeks post-fracture in Lepr^db/+^+SS, Lepr^db/+^+Mg, Lepr^db/db^ + SS, and Lepr^db/db^ + Mg groups. Scale bars, 1 mm. (B) Representative H&E staining of callus at 4- and 8-weeks post-fracture in Lepr^db/+^+SS, Lepr^db/+^+Mg, Lepr^db/db^ + SS, and Lepr^db/db^ + Mg groups. Scale bars, 1 mm. Representative micro-CT images (C) and quantification (D) of BV and BV/TV of the callus at 2 weeks post-fracture in Lepr^db/+^+SS, Lepr^db/+^+Mg, Lepr^db/db^ + SS, and Lepr^db/db^ + Mg groups. ∗p < 0.05, ∗∗p < 0.01, ∗∗∗p < 0.001, by two-way ANOVA with Tukey's *post-hoc* test. Data are presented as mean ± SD. n = 8 per group. Scale bars, 1 mm. Representative micro-CT images (E) and quantification (F) of BV and BMD of the callus at 4 weeks post-fracture in Lepr^db/+^+SS, Lepr^db/+^+Mg, Lepr^db/db^ + SS, and Lepr^db/db^ + Mg groups. ∗∗p < 0.01, ∗∗∗p < 0.001, by two-way ANOVA with Tukey's *post-hoc* test. Data are presented as mean ± SD. n = 8 per group. Scale bars, 1 mm. Representative micro-CT images (G) and quantification (H) of BV and BV/TV of the callus at 8 weeks post-fracture in Lepr^db/+^+SS, Lepr^db/+^+Mg, Lepr^db/db^ + SS, and Lepr^db/db^ + Mg groups. ∗p < 0.05, by two-way ANOVA with Tukey's *post-hoc* test. Data are presented as mean ± SD. n = 8 per group. Scale bars, 1 mm. (I) Four-point bending mechanical test of maximum load, stiffness, and energy to failure of the fractured femur at 8 weeks post-fracture in Lepr^db/+^+SS, Lepr^db/+^+Mg, Lepr^db/db^ + SS, and Lepr^db/db^ + Mg groups. ∗p < 0.05, ∗∗p < 0.01, ∗∗∗p < 0.001, by two-way ANOVA with Tukey's *post-hoc* test. Data are presented as mean ± SD. n = 8 per group.

To evaluate the therapeutic potential of Mg-IMN in T2D fracture healing, we performed histological staining to assess the healing progress of the four groups at weeks 2, 4, and 8 (Fig. 2A and B). H&E and Safranin O/Fast Green staining of callus at week 2 showed larger callus formation in the Lepr^db/+^+Mg group with notably greater woven bone deposition compared to the Lepr^db/+^+SS group (Fig. 2A). Callus in the Lepr^db/db^ + Mg group showed more cartilage formation compared to the Lepr^db/db^ + SS group (Fig. 2A). At week 4, the Lepr^db/+^+Mg group maintained its superior callus development compared to the Lepr^db/+^+SS group, while the diabetic Lepr^db/db^ + Mg group showed significantly advanced callus remodeling than the Lepr^db/db^ + SS group (Fig. 2B). At week 8, the Lepr^db/db^ + SS group exhibited persistent callus retention, whereas the Lepr^db/db^ + Mg group achieved more complete remodeling (Fig. 2B). These findings demonstrate that Mg-IMN treatment promotes early callus formation and accelerates subsequent remodeling in the diabetic fracture healing process, suggesting a dual-phase therapeutic effect that enhances both anabolic and catabolic processes during bone fracture repair.

Micro-CT results at week 2 indicated significantly greater BV and BV/TV in the Lepr^db/+^+Mg group compared to the Lepr^db/+^+SS group and higher BV/TV and TMD in the Lepr^db/+^+SS group compared to the Lepr^db/db^ + SS group (Fig. 2C and D, Supplementary Fig. 2A). At week 4, the Lepr^db/+^+Mg group maintained higher BV and TV, but lower BV/TV compared to the Lepr^db/+^+SS group. The Lepr^db/db^ + SS group displayed increased TV but impaired mineralization, as evidenced by significantly lower BV/TV and BMD compared to the Lepr^db/+^+SS group. Notably, Mg-IMN treatment partially rescued this phenotype, with the Lepr^db/db^ + Mg group showing improved BMD compared to the Lepr^db/db^ + SS group (Fig. 2E and F, Supplementary Fig. 2B). The therapeutic effects became more pronounced at week 8, with the Lepr^db/db^ + Mg group demonstrating superior BV and BV/TV compared to the Lepr^db/db^ + SS group. Persistent mineralization deficits were observed in the Lepr^db/db^ + SS group, as shown by significantly reduced BV/TV and TMD in the Lepr^db/db^ + SS group compared to the Lepr^db/+^+SS group at week 8 (Fig. 2G and H, Supplementary Fig. 2C). These results suggested that the promotion effect of Mg-IMN on T2D diabetic fracture healing started to occur in the middle stage of the process and enhanced callus reconstruction in the later regenerative stage. The consistent patterns observed between the Lepr^db/+^+SS and Lepr^db/+^+Mg groups corroborate our previous findings on the osteogenic properties of Mg in normal fracture repair. Furthermore, the four-point bending biomechanical test at week 8 showed lower maximum load, stiffness, and energy to failure in the Lepr^db/db^ + SS group compared to the Lepr^db/+^+SS group and a significantly increased maximum load, stiffness, and energy to failure in the Lepr^db/db^ + Mg group compared to the Lepr^db/db^ + SS group (Fig. 2I), suggesting that T2D significantly compromises fracture healing capacity, while Mg-IMN intervention effectively restores mechanical properties to near-normal levels.

To further investigate the role of Mg in bone metabolism, especially cortical bone homeostasis, we conducted the Mg-IMN implantation surgery in the right femur without fracture in Lepr^db/+^ and Lepr^db/db^ mice. Femora were harvested bilaterally for all four groups 4 weeks after surgery. Micro-CT analysis of the right femur of the four groups showed increased BV and cortical thickness (Ct.Th) after Mg-IMN implantation in both Lepr^db/+^ +Mg-UF and Lepr^db/db^ + Mg-UF groups (Supplementary Fig. 3A and B). Both in the Lepr^db/+^ and Lepr^db/db^ mice, the BV and Ct.Th of the left femur showed no significant difference with or without Mg-IMN implantation in the right femur (Supplementary Fig. 3C and D). These results indicate that the promotive effect of Mg-IMN on new bone formation mainly occurs in the local environment and does not systemically affect contralateral femora.

### 3.3 Mg-IMN increases Treg infiltration in callus

**Fig. 3:**
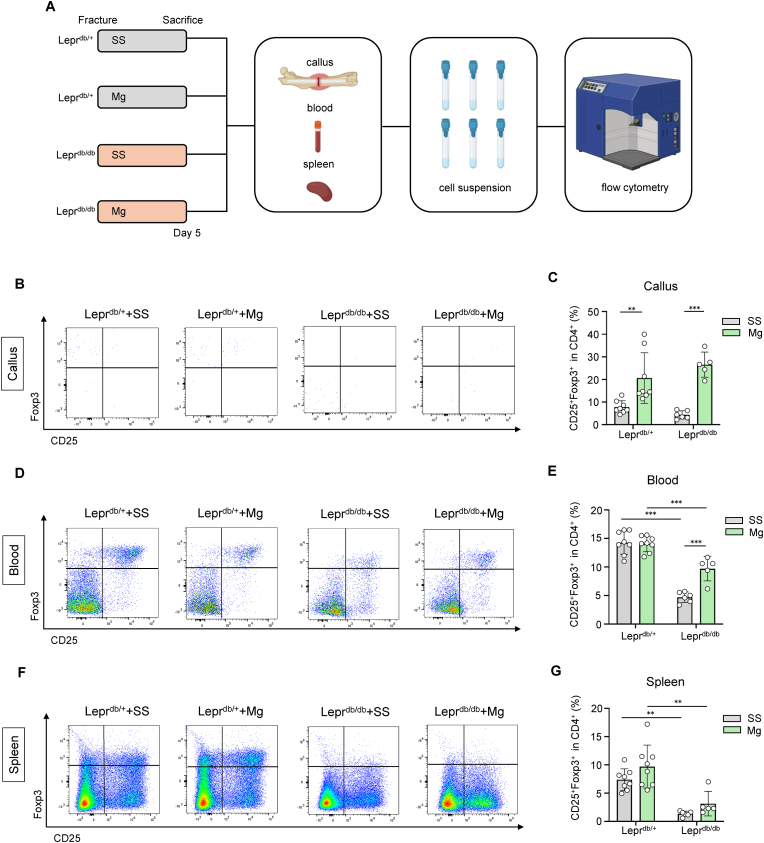
**Mg-IMN increases Treg infiltration in callus in both Lepr^db/+^ and Lepr^db/db^ mice.** (A) Schematic diagram showing the experimental design of flow cytometry. Representative flow cytometry plots (B) and frequency (C) of CD25^+^Foxp3^+^ Tregs in callus at 5 days post-fracture in Lepr^db/+^+SS, Lepr^db/+^+Mg, Lepr^db/db^ + SS, and Lepr^db/db^ + Mg groups. ∗∗p < 0.01, by two-way ANOVA with Tukey's *post-hoc* test. Data are presented as mean ± SD. n = 5-8 per group. Representative flow cytometry plots (D) and frequency (E) of CD25^+^Foxp3^+^ Tregs in blood at 5 days post-fracture in Lepr^db/+^+SS, Lepr^db/+^+Mg, Lepr^db/db^ + SS, and Lepr^db/db^ + Mg groups. ∗∗p < 0.01, ∗∗∗p < 0.001, by two-way ANOVA with Tukey's *post-hoc* test. Data are presented as mean ± SD. n = 5-8 per group. Representative flow cytometry plots (F) and frequency (G) of CD25^+^Foxp3^+^ Tregs in the spleen at 5 days post-fracture in Lepr^db/+^+SS, Lepr^db/+^+Mg, Lepr^db/db^ + SS, and Lepr^db/db^ + Mg groups. ∗∗p < 0.01, ∗∗∗p < 0.001, by two-way ANOVA with Tukey's *post-hoc* test. Data are presented as mean ± SD. n = 5-8 per group.

The changes of Tregs after Mg-IMN implantation were explored in the Lepr^db/+^ and Lepr^db/db^ mice at 5 days post-fracture (Fig. 3A). The implantation of Mg-IMN significantly increased the frequency of Tregs in callus both in the Lepr^db/+^+Mg group and the Lepr^db/db^ + Mg group (Fig. 3B and C). The enrichment of Tregs in the callus after Mg-IMN implantation suggests the potential role of Tregs during the fracture healing process. While in blood, the higher frequency of Tregs was just observed in the Lepr^db/db^ + Mg group compared to the Lepr^db/db^ + SS group, with no significant difference between the Lepr^db/+^+ SS group and Lepr^db/+^+ Mg group (Fig. 3D and E). Decreased Tregs in the spleen were observed in the Lepr^db/db^ + SS group compared to the Lepr^db/+^+SS group (Fig. 3F and G). No significant change in the frequency of Tregs in the spleen between the Lepr^db/+^+SS and Lepr^db/+^+Mg groups or the Lepr^db/db^ + SS and Lepr^db/db^ + Mg groups. These results indicate the complex interaction between Mg-IMN implantation and immune regulation in the process of diabetic fracture healing.

### 3.4 Treg depletion does not result in changes in T2D conditions in Lepr^db/db^ mice

**Fig. 4:**
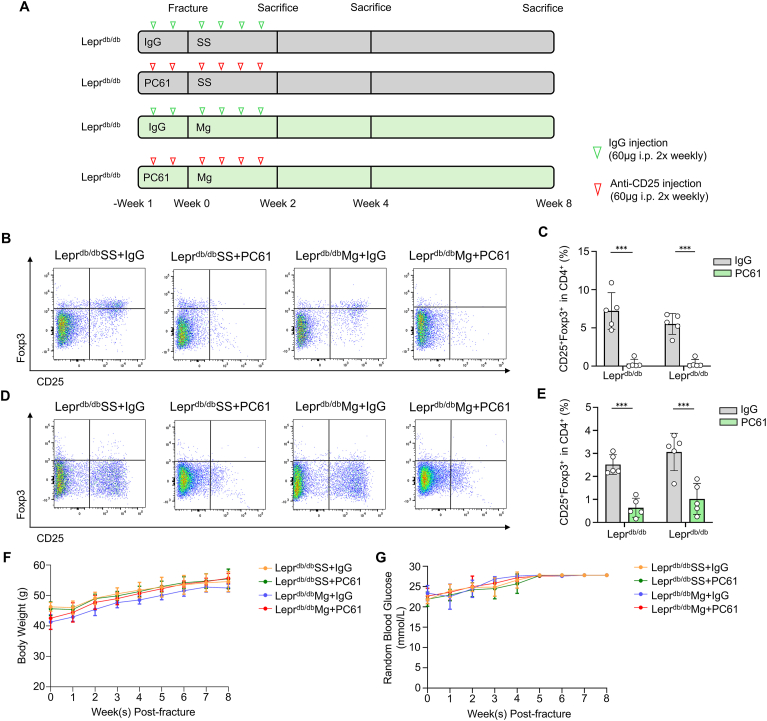
**Treg depletion does not result in a T2D change in Lepr^db/db^ mice.** (A) Schematic diagram showing the experimental design and timeline of Treg depletion and closed femoral fracture in Lepr^db/db^ mice. Representative flow cytometry plots (B) and frequency (C) of CD25^+^Foxp3^+^ Tregs in blood at 2 weeks post-fracture in Lepr^db/db^SS + IgG, Lepr^db/db^SS + PC61, Lepr^db/db^Mg + IgG, and Lepr^db/db^Mg + PC61 groups. ∗∗∗p < 0.001, by two-way ANOVA with Tukey's *post-hoc* test. Data are presented as mean ± SD. n = 5 per group. Representative flow cytometry plots (D) and frequency (E) of CD25^+^Foxp3^+^ Tregs in the spleen at 2 weeks post-fracture in Lepr^db/db^SS + IgG, Lepr^db/db^SS + PC61, Lepr^db/db^Mg + IgG, and Lepr^db/db^Mg + PC61 groups. ∗∗∗p < 0.001, by two-way ANOVA with Tukey's *post-hoc* test. Data are presented as mean ± SD. n = 5 per group. (F) Comparison of the body weight between Lepr^db/db^SS + IgG, Lepr^db/db^SS + PC61, Lepr^db/db^Mg + IgG, and Lepr^db/db^Mg + PC61 groups from 0 to 8 weeks post-fracture. n = 5 per group. (G) Comparison of the random blood glucose between Lepr^db/db^SS + IgG, Lepr^db/db^SS + PC61, Lepr^db/db^Mg + IgG, and Lepr^db/db^Mg + PC61 groups from 0 to 8 weeks post-fracture. n = 5 per group.

To investigate the essential role of Tregs in mediating the beneficial effects of Mg-IMN on fracture healing in T2D mice, we performed functional Treg depletion using anti-CD25 monoclonal antibodies (clone PC61). The depletion protocol consisted of intraperitoneal injections administered twice weekly, beginning one week before fracture surgery and continuing for two weeks post-fracture, with control group receiving equivalent doses and frequency of IgG isotype antibody (Fig. 4A). Flow cytometric analysis confirmed the specific and efficient depletion of Tregs in both blood (Fig. 4B and C, Supplementary Fig. 4A) and spleen (Fig. 4D and E, Supplementary Fig. 4B) of Lepr^db/db^ mice. To exclude the potential effect of Treg depletion on systemic T2D conditions, we monitored body weight and random blood glucose levels in four experimental groups: Lepr^db/db^SS + IgG, Lepr^db/db^SS + PC61, Lepr^db/db^Mg + IgG, and Lepr^db/db^Mg + PC61 (Fig. 4F and G). Notably, no significant differences in body weight or random blood glucose levels were observed between the Lepr^db/db^Mg + IgG and Lepr^db/db^Mg + PC61 groups. These findings demonstrate that the observed biological effects following Treg depletion are specifically mediated through modulation of local responses rather than systemic changes in diabetic conditions, thereby establishing Tregs as crucial mediators of Mg-IMN-enhanced fracture healing in T2D mice.

### 3.5 Treg depletion impairs the effect of Mg-IMN on promoting diabetic fracture healing

**Fig. 5:**
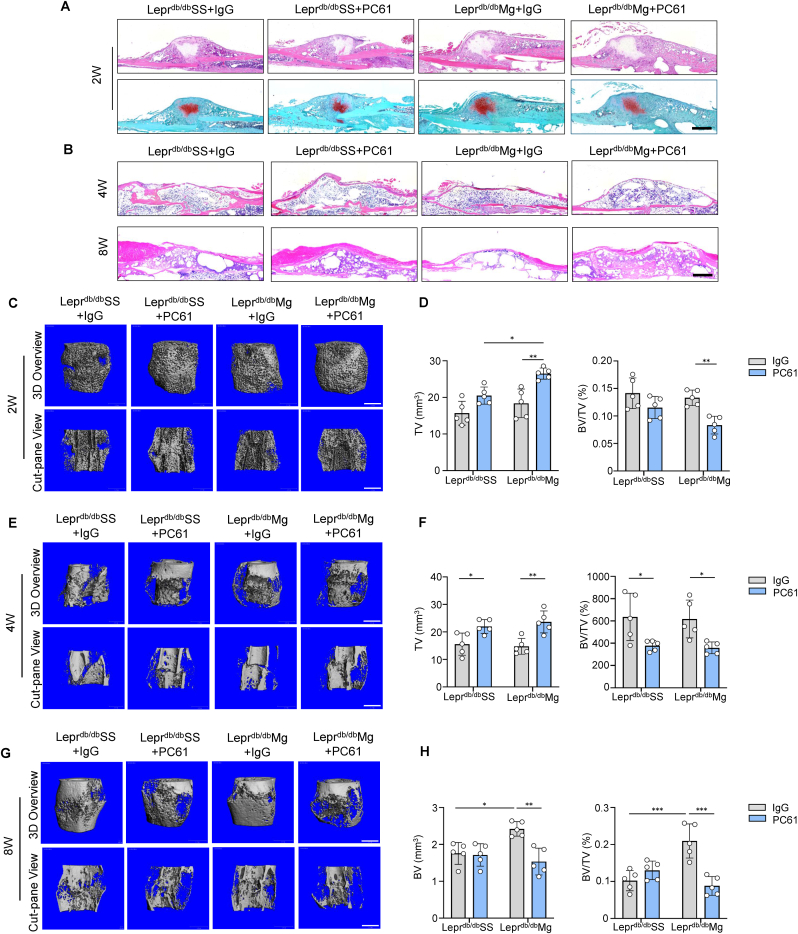
**Treg depletion impairs the effect of Mg-IMN on promoting T2D diabetic fracture healing.** (A) Representative H&E and Safranin O/Fast Green staining of callus at 2 weeks post-fracture in Lepr^db/db^SS + IgG, Lepr^db/db^SS + PC61, Lepr^db/db^Mg + IgG, and Lepr^db/db^Mg + PC61 groups. Scale bars, 1 mm. (B) Representative H&E staining of callus at 4- and 8-weeks post-fracture in Lepr^db/db^SS + IgG, Lepr^db/db^SS + PC61, Lepr^db/db^Mg + IgG, and Lepr^db/db^Mg + PC61 groups. Scale bars, 1 mm. Representative micro-CT images (C) and quantification (D) of TV and BV/TV of the callus at 2 weeks post-fracture in Lepr^db/db^SS + IgG, Lepr^db/db^SS + PC61, Lepr^db/db^Mg + IgG, and Lepr^db/db^Mg + PC61 groups. ∗p < 0.05, ∗∗p < 0.01, by two-way ANOVA with Tukey's *post-hoc* test. Data are presented as mean ± SD. n = 5 per group. Scale bars, 1 mm. Representative micro-CT images (E) and quantification (F) of TV and BV/TV of the callus at 4 weeks post-fracture in Lepr^db/db^SS + IgG, Lepr^db/db^SS + PC61, Lepr^db/db^Mg + IgG, and Lepr^db/db^Mg + PC61 groups. ∗p < 0.05, ∗∗p < 0.01, by two-way ANOVA with Tukey's *post-hoc* test. Data are presented as mean ± SD. n = 5 per group. Scale bars, 1 mm. Representative micro-CT images (G) and quantification (H) of BV and BV/TV of the callus at 8 weeks post-fracture in Lepr^db/db^SS + IgG, Lepr^db/db^SS + PC61, Lepr^db/db^Mg + IgG, and Lepr^db/db^Mg + PC61 groups. ∗p < 0.05, ∗∗p < 0.01, ∗∗∗p < 0.001, by two-way ANOVA with Tukey's *post-hoc* test. Data are presented as mean ± SD. n = 5 per group. Scale bars, 1 mm.

To explore the effect of Treg depletion on Mg-IMN-promoting T2D diabetic fracture healing, we performed histological staining of the callus at weeks 2, 4, and 8 (Fig. 5A and B). H&E and Safranin O/Fast Green staining of the callus at week 2 revealed larger callus formation in the Lepr^db/db^Mg + PC61 group compared to the Lepr^db/db^Mg + IgG group (Fig. 5A). Additionally, the Lepr^db/db^Mg + IgG group exhibited better cartilage formation than the Lepr^db/db^SS + IgG group (Fig. 5A), consistent with our previous findings. At week 4, the Lepr^db/db^Mg + PC61 group maintained a larger callus than the Lepr^db/db^Mg + IgG group; also, the larger callus formation in the Lepr^db/db^ SS + PC61 group compared to the Lepr^db/db^SS + IgG group (Fig. 5B). At week 8, the Lepr^db/db^Mg + IgG group showed superior callus remodeling than the Lepr^db/db^Mg + PC61 group (Fig. 5B), suggesting that the injection of PC61 led to impaired callus reconstruction of the Lepr^db/db^ Mg + PC61 group. Collectively, these findings indicate that Treg depletion not only negates the beneficial effects of Mg-IMN on diabetic fracture healing but also exacerbates impaired healing under normal T2D conditions.

Micro-CT analysis revealed impaired fracture healing following Treg depletion across all time points. At week 2, the Lepr^db/db^Mg + PC61 group showed higher TV and lower BV/TV compared to the Lepr^db/db^Mg + IgG group (Fig. 5C and D), along with increased Tb.Sp and decreased Tb.N compared to the Lepr^db/db^Mg + IgG group (Supplementary Fig. 5A). These results suggest abnormally enlarged and decreased woven bone formation of the callus after Tregs depletion at week 2. At week 4, the Lepr^db/db^Mg + PC61 group showed higher TV and lower BV/TV compared to the Lepr^db/db^Mg + IgG group (Fig. 5E and F). The Lepr^db/db^SS + PC61 group showed higher TV and lower BV/TV compared to the Lepr^db/db^SS + IgG group (Fig. 5E). At week 8, the Lepr^db/db^Mg + IgG group showed higher BV, BV/TV, BMD, and TMD compared to the Lepr^db/db^Mg + PC61 group (Fig. 5G and H, Supplementary Fig. 5B). The Lepr^db/db^Mg + PC61 group showed increased TV compared to the Lepr^db/db^Mg + IgG group (Supplementary Fig. 5B). The Lepr^db/db^Mg + IgG group also showed increased BV, BV/TV, BMD and TMD compared to the Lepr^db/db^SS + IgG group (Fig. 5G and Supplementary Fig. 5B). These results suggested that the depletion of Tregs impaired bone fracture healing from early stage to late stage under T2D conditions. The promotion effect of Mg-IMN on T2D diabetic fracture healing was further supported by the comparison between Lepr^db/db^SS + IgG and Lepr^db/db^Mg + IgG groups.

### 3.6 Bulk RNA sequencing of the callus from the Lepr^db/db^ + SS and Lepr^db/db^ + Mg groups at five days post-fracture

**Fig. 6:**
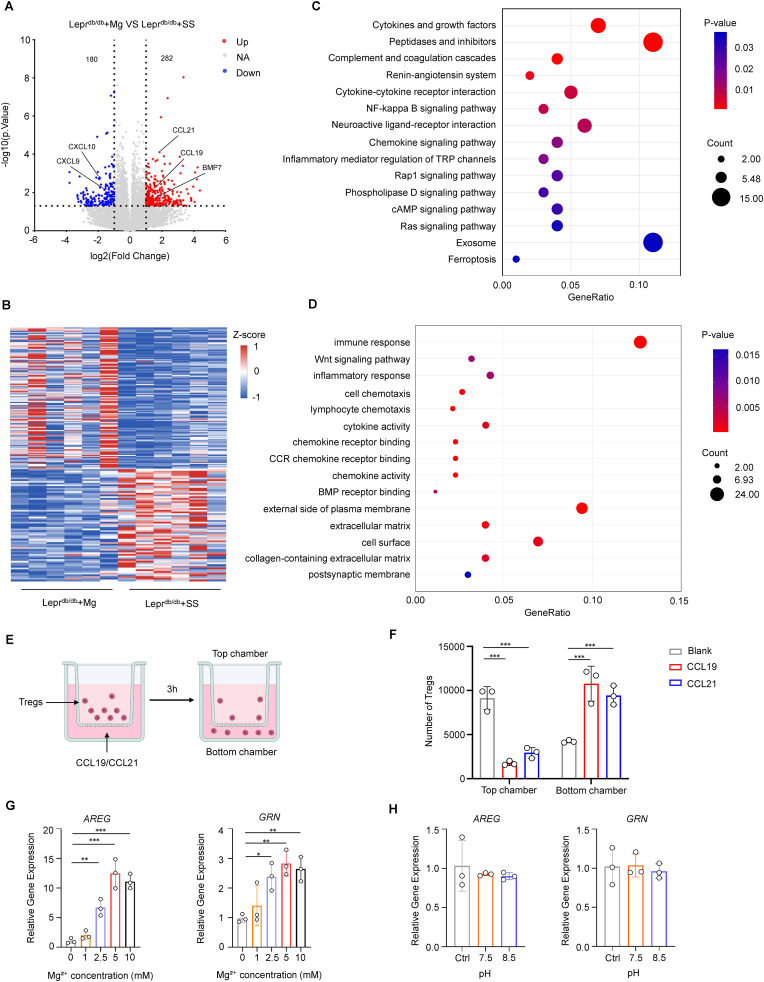
**Bulk RNA sequencing of the callus from the Lepr^db/db^** + **SS and Lepr^db/db^** + **Mg groups at 5 days post-fracture**. (A-D) Bulk RNA sequencing of the callus from the fractured femur of the Lepr^db/db^ + SS and Lepr^db/db^ + Mg groups. DEGs were set as |log2 fold change| > 1 and p < 0.05; calluses from two mice were combined as one sample for bulk RNA sequencing, with a total of twelve mice analyzed for each group. (A) Volcano plots of DEGs of the callus from the Lepr^db/db^ + SS and Lepr^db/db^ + Mg groups at 5 days post-fracture. (B) Heatmap illustrating the expression patterns of DEGs in callus from the Lepr^db/db^ + SS (right columns) and Lepr^db/db^ + Mg (left columns) groups. (C) KEGG pathway enrichment analysis of up-regulated and down-regulated DEGs of the callus from the Lepr^db/db^ + SS and Lepr^db/db^ + Mg groups at 5 days post-fracture. (D) GO enrichment analysis of up-regulated and down-regulated DEGs of the callus from the Lepr^db/db^ + SS and Lepr^db/db^ + Mg groups at 5 days post-fracture. (E) Schematic diagram showing the CCL19/CCL21-mediated chemotaxis assay protocol. (F) The number of Tregs in the top/bottom chambers treated with CCL19/CCL21 at 20 ng/mL ∗∗∗p < 0.001, by two-way ANOVA with Bonferroni's multiple-comparisons test. Data are presented as mean ± SD. n = 3 per group. (G) Relative mRNA expression of *AREG* and *GRN* in Tregs under different concentrations of Mg^2+^ treatment. ∗p < 0.05, ∗∗p < 0.01, ∗∗∗p < 0.001, by unpaired two-tailed Student's *t*-test. Data are presented as mean ± SD. n = 3 per group. (H) Relative mRNA expression of *AREG* and *GRN* in Tregs cultured at different pH levels. By unpaired two-tailed Student's *t*-test. Data are presented as mean ± SD. n = 3 per group.

To further explore the potential mechanisms by which Tregs contribute to Mg-IMN-enhanced fracture healing in T2D, Bulk RNA sequencing was performed on callus isolated from the Lepr^db/db^ + SS and Lepr^db/db^ + Mg groups at 5 days post-fracture. The analysis identified 282 significantly upregulated and 180 significantly downregulated differentially expressed genes (DEGs) in the Lepr^db/db^ + Mg group compared to the Lepr^db/db^ + SS group (Fig. 6A). A heatmap illustrated the upregulated and downregulated DEGs in the Lepr^db/db^ + Mg group relative to the Lepr^db/db^ + SS group (Fig. 6B). We found that after Mg-IMN implantation, the genes *Ccl19* and *Ccl21*, which are associated with chemokine activity, and *Bmp7*, linked to osteogenesis, were upregulated (Fig. 6B). Conversely, genes like *Cxcl9* and *Cxcl10*, which are involved in T cell chemotaxis and inflammation, were downregulated in the Lepr^db/db^ + Mg group compared to the Lepr^db/db^ + SS group (Fig. 6B). These findings suggest that Mg-IMN may facilitate diabetic fracture healing by regulating osteogenesis-related genes while downregulating inflammation-related genes. Additionally, chemokines CCL19 or CCL21 may serve as potential targets for the extensive recruitment of Tregs in the callus following Mg-IMN implantation, warranting further exploration.

Pathway analysis using the Kyoto Encyclopedia of Genes and Genomes (KEGG) revealed significant enrichment in pathways related to cytokines and growth factors, cytokine-cytokine receptor interaction, and chemokine signaling pathway after Mg-IMN was implanted (Fig. 6C). Gene Ontology (GO) enrichment analyses were also performed to investigate the biological functions and pathways of the DEGs (Fig. 6D). In terms of biological process (BP), DEGs were significantly enriched in immune response, Wnt signaling pathway, inflammatory response, cell and lymphocyte chemotaxis (Fig. 6D). For molecular function (MF), DEGs were significantly enriched in cytokine activity, chemokine receptor binding, CCR chemokine receptor binding, chemokine activity and BMP receptor binding (Fig. 6D). These findings suggest that Mg-IMN implantation triggers a corresponding immune response and upregulation of osteogenesis within the callus. The significant enrichment of chemokine signaling pathways may also account for the increased presence of Tregs in the callus. However, specific pathways and functions still need further investigation.

To assess the chemotactic potential of CCL19 and CCL21 in recruiting Tregs, we conducted transwell migration assays *in vitro* (Fig. 6E). Flow cytometric analysis revealed that both CCL19 and CCL21 significantly enhanced Treg migration across the transwell membrane (Fig. 6F), indicating their chemotactic effects on Treg trafficking. Subsequently, we examined the modulatory effect of three degradation products of Mg-IMN on the function of Tregs. Following a 5-day differentiation period, Tregs were treated with a gradient of Mg^2+^ concentrations and subsequently co-cultured for 24 h qRT-PCR analysis demonstrated that Mg^2+^ treatment significantly upregulated the expression of genes *Areg* and *Grn* in Tregs in a dose-dependent manner (Fig. 6G), both of which have been reported to facilitate bone fracture repair [21,22]. While the mRNA expression levels of key Treg-associated markers (*FOXP3*, *CD25*, *IL-10*, and *CTLA-4*) showed no significant differences between Mg^2+^-treated and control group (Supplementary Fig. 6A), indicating that Mg^2+^ administration does not substantially affect the transcriptional profiles of canonical Treg signature genes under the present experimental conditions. To assess whether the alkaline microenvironment resulting from Mg-IMN degradation influences Treg function, we treated *in vitro*-differentiated Tregs with media adjusted to different pH values according to our previosue study [13], followed by qRT-PCR analysis. The results showed that the alkaline environment generated by the degradation of Mg-IMN did not significantly affect the function of Tregs (Fig. 6G and Supplementary Fig. 6B). We further explored the potential impact of hydrogen on Tregs by culturing Tregs in H_2_-rich medium for 24 h. No significant changes in Treg functional markers were observed following hydrogen exposure (Supplementary Fig. 6C). Likewise, exposure to high glucose conditions for 24 h also failed to produce any notable change in Treg function, as determined by qRT-PCR (Supplementary Fig. 6D).

## 4 Discussion

In the current study, we investigated the role of Mg-IMN in enhancing fracture healing in the condition of T2D and explored the potential role of Tregs in this process. Our findings demonstrate that Mg-IMN not only accelerates fracture healing in T2D mice but also significantly improves the quality of the callus. We found an increase in Treg frequency within the callus by day 5 post-fracture after Mg-IMN implantation. Importantly, depletion of Tregs through PC61 antibody injection completely abolished the promotive effects of Mg-IMN, highlighting the critical role of Tregs in this healing process. Furthermore, bulk RNA sequencing of the callus at 5 days identified significant enrichment of pathways associated with chemokine receptor binding, CCR chemokine receptor interactions, and chemokine signaling, which may contribute to the recruitment of Tregs. Our *in vitro* investigation further indicated that Tregs are recruited to the fracture site in response to the CCL19/CCL21-CCR7 axis after Mg-IMN implantation. This recruitment promotes bone fracture healing by enhancing the secretion of Amphiregulin (AREG) and progranulin (PGRN), both of which are known to be secreted by Tregs to promote the healing process.

### 4.1 Mg-IMN promotes bone fracture healing and new bone formation in T2D Lepr^db/db^ mice

Increased fracture risk and fracture non-union are serious complications of T2D [30,31]. The complex bone structure changes caused by various pathogenic factors in patients with T2D make the conventional dual-energy X-ray absorptiometry underestimate the fracture risk prediction of T2D [32]. Although patients with T2D display a unique skeletal phenotype with normal or even increased BMD, the structural and geometric properties of their bones are impaired due to altered collagen arrangement caused by the accumulation of AGEs, which collectively contribute to increased bone fragility and heightened fracture susceptibility [32]. Meanwhile, decreased osteoblast activity and unchanged or slightly increased osteoclast activity, leading to low bone turnover under T2D conditions, contribute to impaired bone fracture healing [33]. To further enrich the therapeutic context of bone regeneration and address the challenges highlighted in recent explorations [[34], [35], [36], [37]], novel strategies specifically targeting diabetic fracture are highly warranted. Our results indicated that Mg-IMN significantly facilitates the remodeling phase of fracture callus in the Lepr^db/db^ T2D mice model. Specifically, Mg-IMN not only accelerated the overall fracture healing process but also markedly improved the structural integrity and mechanical strength of the newly formed callus tissue. These results suggest that Mg-IMN may serve as an effective therapeutic strategy to address impaired bone regeneration commonly observed in diabetic conditions, thereby enhancing both the quality and functionality of fracture repair in T2D. Additionally, our SS or Mg-IMN implantation experiments indicated that the promotive effects of Mg-IMN on new bone formation are localized and do not systemically affect the contralateral femora.

### 4.2 Changes in frequencies of Tregs across tissues after Mg-IMN implantation

Bone fracture healing is a continuous and delicate process which divided into four stages: hematoma formation, soft callus formation, hard callus formation, callus remodeling and reconstruction [38]. When a fracture occurs, various immune cells are recruited to the fracture site and initiate the repair process [39]. Among the immune cells involved, Tregs are gaining increasing attention due to their unique immunosuppressive effects. This unique subset of CD4^+^ T cells, accounting for only 5-10% of total CD4^+^ T cells, but play a crucial role in maintaining immune homeostasis [40]. Clinical studies have indicated that patients treated with immunosuppressive agents exhibit delayed bone healing and a higher incidence of non-healing bones in HIV patients [39]. Similarly, in C57 mice, depletion of Tregs after fracture impairs bone fracture healing [22]. In recent years, studies on Tregs in fracture healing have found that Tregs are enriched in the fracture callus in the early stage (5 or 7 days post-fracture) of fracture healing [21,22]. Our study showed that while T2D mice implanted with the stainless-steel nails (SS) showed reduced Tregs in the blood and spleen compared to normal mice implanted with SS, T2D mice fixed with Mg-IMN showed high levels of Tregs in the callus, comparable to those of normal mice fixed with Mg-IMN. We hypothesize that the observed differences in Treg dynamics between the fracture callus and blood are primarily attributable to the distinct recruitment effects exerted by the varying fracture microenvironments. The enrichment of Tregs in the fracture callus 5 days after Mg-IMN implantation implies a potential regulatory role of Tregs in Mg-IMN-mediated fracture healing. This result suggests that the implantation of Mg-IMN, whether in normal or diabetic mice, alters the fracture microenvironment compared to fractures fixed with SS. The distinct fracture microenvironments created by Mg-IMN through Mg degradation after implantation may exert similar recruitment effects on Tregs during fracture healing, thereby promoting fracture healing. However, whether the recruitment pathway of Tregs is the same across different fracture microenvironments requires further investigation. Meanwhile, there is no significant change in the frequencies of Tregs in the spleen between the Lepr^db/db^ + SS and Lepr^db/db^ + Mg groups, suggesting a potential influence of Mg-IMN on the distribution and maintenance of Tregs in secondary lymphoid organs. Meanwhile, the frequency of Tregs in blood 5 days after fracture in the Lepr^db/db^ + Mg group was higher than the Lepr^db/db^ + SS group, while the Lepr^db/+^+Mg group showed a similar level as the Lepr^db/+^+SS group. We propose that this difference arises from a compensatory increase in the local recruitment of impaired Treg populations in T2D, responding to the altered fracture microenvironment created by Mg-IMN implantation.

### 4.3 Treg depletion abolishes the positive effects of Mg-IMN on promoting fracture healing in diabetic mice

To further investigate the role of Tregs in this process, we administrated PC61 via intraperitoneal injection to deplete Tregs in Lepr^db/db^ mice. The results indicated that after Treg depletion, the fracture callus in the Lepr^db/db^Mg + PC61 group showed increased TV and decreased BV/TV from early to late stages of fracture healing, highlighting the role of Tregs in bone remodeling. By week 8, the callus in the Lepr^db/db^Mg + PC61 group showed impaired healing similar to that in the Lepr^db/db^SS + IgG group, indicating the promotive effects of Mg-IMN on diabetic fracture healing nearly completely disappeared after Treg depletion. These results underscore the essential role of Tregs in this healing process. While depletion of regulatory T cells (DEREG) mice enable specific and inducible Treg depletion [41], they are less suitable for modeling T2D than Lepr^db/db^ mice. Inducing a T2D phenotype in DEREG mice typically requires a high-fat diet and streptozotocin (STZ) injections, which increase experimental complexity and may compromise model stability. Furthermore, although classic depletion methods like anti-CD25 antibodies are relatively cost-effective [42], they suffer from limited specificity and incomplete Treg clearance. Similarly, conventional constitutive DTR mouse models often lead to persistent immune dysfunction and/or developmental abnormalities, which can confound the analysis of fracture healing. Even with these advanced strategies, complete Treg depletion remains challenging because Tregs may repopulate [43]. In contrast, the Lepr^db/db^ mice model used in this study spontaneously develops typical hyperglycemia, insulin resistance, and bone metabolic disorders without exogenous chemical induction. This provides a pathological model that closely reflects the clinical features of T2D while avoiding the confounding effects induced by complex modeling procedures. Therefore, investigating Treg depletion within the Lepr^db/db^ background provides more physiologically relevant and reliable insights into diabetic fracture healing.

### 4.4 Further exploration of the underlying mechanisms involved in Treg recruitment and their role in promoting diabetic fracture repair

To further explore the mechanism of Treg recruitment and its effect on fracture healing, bulk RNA sequencing of the callus from the Lepr^db/db^ + SS and Lepr^db/db^ + Mg groups was performed. Analysis of the sequencing results showed that after Mg-IMN implantation, genes related to *Ccl19*, *Ccl21*, and *Bmp7* in the callus were significantly upregulated. CCL19 has been reported to have a strong effect on the migration ability of Tregs [44]. *In vitro* experiments showed that Tregs exhibited enhanced homing ability after stimulation with the CCR7 ligand CCL21, which upregulated CCR7 expression [45]. Furthermore, the CCL19/CCL21-CCR7 signaling axis has been demonstrated to be essential for preserving the immunosuppressive function of Tregs [46]. These results provide insights into the recruitment of Tregs to the fracture callus following Mg-IMN implantation. Apart from the CCL19/CCL21-CCR7 axis, Mg^2+^-primed M2 macrophages and MSCs interact with Tregs. M2 macrophages secrete TGF-β and IL-10 to stabilize Tregs through STAT3/SMAD3 signaling [47,48], whereas Mg-stimulated MSCs elevate ICOS-L and PD-L1 to strengthen Treg immunosuppression [49]; meanwhile, the endothelial TRPM7-NFAT5 cascade drives angiogenesis and Treg recruitment [49]. In addition, Tregs coordinate fracture repair via broad intercellular interactions with various cells in the fracture microenvironment. Tregs in fracture callus secrete high levels of AREG, which activates the EGFR-PI3K/AKT pathway in osteoprogenitors to enhance their proliferation and osteogenic differentiation into mature osteoblasts [22]. Tregs drive M1-to-M2 macrophage polarization via IL-10 secretion and strengthen the TGF-β/IL-10-mediated anti-inflammatory capacity to facilitate bone regeneration [39]. These cell-cell crosstalk events form a coordinated osteoimmune network that shifts local inflammation toward tissue regeneration, offering promising molecular targets for diabetic fracture treatment with Mg-IMN implants. Additionally, BMP7 has been widely shown to effectively promote fracture healing [50]. Pathway analysis using KEGG and GO enrichment analyses revealed that DEGs were significantly enriched in immune activity pathways such as the chemokine signaling pathway, CCR chemokine receptor binding, and BMP receptor binding. In summary, after Mg-IMN implantation under T2D conditions, Tregs are likely to be recruited to the fracture callus in response to the activation of CCR receptors, contributing to the promotion of diabetic fracture healing. We preliminarily verified this hypothesis via *in vitro* experiments.

The transwell assay results showed that both CCL19 and CCL21 promoted Treg migration. Moreover, Tregs treated with Mg^2+^ expressed higher levels of *Areg* and *Grn*, which have been reported to be secreted by Tregs and to promote bone fracture healing in previous studies [21,22]. The mRNA expression levels of key Treg-associated markers (*FOXP3*, *CD25*, *IL-10*, and *CTLA-4*) showed no significant differences between Mg^2+^-treated and control groups, indicating that Mg^2+^ supplementation does not markedly alter the transcriptional expression of canonical Treg signature genes under the experimental conditions. In addition, our current results indicated that other degradation products of Mg-IMN, such as hydrogen and the alkaline microenvironment, as well as the high-glucose condition, did not affect the intrinsic function of Tregs or alter their osteogenic potential under the present experimental conditions.

In summary, our findings prove that Mg-IMN promotes fracture healing in T2D by recruiting more Tregs into the callus. However, it is important to note that this recruitment effect is not directly caused by the degradation of products (Mg^2+^, H_2_, or OH^−^) of the Mg compartment in Mg-IMN. Although these degradation products have been shown to promote fracture healing through various mechanisms [12,13], there are currently no research results indicating that they directly influence Treg recruitment. The impact of Mg-IMN on the fracture microenvironment is also crucial. Under normal physiological conditions and in various diseases, the recruitment and localization of Tregs are highly dependent on chemokine signals, which serve as a "directional navigation system" that guides their migration [[51], [52], [53]]. According to the current study, the CCL19/CCL21-CCR7 axis is a direct factor that promotes the recruitment of Tregs to the fracture callus. Studies have shown that lymphatic endothelial cells are the primary source of CCL19/CCL21 and play a crucial role in regulating and recruiting Tregs [54,55], providing a direction for our future research.

Although studies have shown that Tregs can be recruited to the fracture site via the CCL1-CCR8 axis to promote fracture repair during the normal fracture healing process [21], this does not conflict with our findings. Instead, it indicates that the use of Mg-IMN to stabilize fractures under T2D conditions creates distinct fracture microenvironments, leading to different recruitment effects on Tregs. Meanwhile, our *in vitro* experiments confirmed that Tregs treated with Mg^2+^ showed significant upregulation of genes encoding AGRE and GRN, both of which have been reported to be secreted by Tregs and are known to promote fracture healing [21,22]. These results indicate that Tregs recruited to the fracture callus can respond to the Mg^2+^ generated by the local degradation of the Mg compartment from Mg-IMN, leading to increased production of AREG and GRN, thereby enhancing fracture repair.

### 4.5 Limitations of the study and future focus of investigations

Despite the promising findings, this study has some limitations. Firstly, our study primarily examined Treg alterations at day 5 post-fracture. This time point cprrespondes to a critical window in which Tregs to exert pro-osteogenic functions during early endochondral ossification. Nevertheless, future investigations should include additional time points across the entire healing process to fully characterize the dynamic profiles and temporal distribution of Tregs following Mg-IMN implantation. Secondly, *in vitro* culture conditions cannot fully recapitulate the diabetic inflammatory microenvironment. The addition of pro-inflammatory factors can markedly interfere with Treg induction and phenotypic maintenance, potentially leading to unreliable or even misleading conclusions. Thirdly, the current study did not further investigate whether Mg^2+^ regulates CCL19/CCL21 directly or indirectly, nor did it identify the cellular sources of these chemokines. In future work, we will conduct both *in vitro* and *in vivo* experiments to identify the key cellular sources and explore the precise signaling pathways, thereby clarifying the mechanistic link between Mg^2+^ and CCL19/CCL21 expression. Lastly, although current *in vitro* results indicate that hydrogen and the alkaline microenvironment generated by Mg-IMN exert no significant influence on Treg function, further *in vivo* investigations of dynamic monitoring of local pH and H_2_ concentrations, together with assessment of their effects on Tregs, will help validate and further strengthen this conclusion.

## Ethics approval and consent to participate

All animal experiment procedures performed in this study adhered to the principles for the care and use of laboratory animals approved by the Animal Experimentation Ethics Committee of the Chinese University of Hong Kong (Ref. No. 22-242-MIS-5-C and 23-174-MIS-5-C).

## Funding

This work was supported by Areas of Excellence (AoE/M-402/20), National Natural Science Foundation of China (82002359), Health and Medical Research Fund (HMRF, 09203786), NSFC/RGC Joint Research Scheme (N_CUHK405/21), Research Impact Fund (R4034-23F), 10.13039/501100004853CUHK Direct Grant (2025.134 and 2026.308), and Guangdong Basic and Applied Basic Research Foundation (Ref No. 2020A1515010267, 2022A1515010510).

## CRediT authorship contribution statement

**Shi'an Zhang:** Conceptualization, Data curation, Formal analysis, Investigation, Methodology, Project administration, Validation, Visualization, Writing – original draft, Writing – review & editing. **Bingyang Dai:** Data curation, Formal analysis, Investigation, Methodology, Software, Validation. **Yuanming An:** Data curation, Formal analysis, Investigation, Methodology, Software, Validation. **Zheyu Jin:** Data curation, Formal analysis, Investigation, Methodology, Software, Validation. **Weiyang Liu:** Data curation, Formal analysis, Investigation, Methodology, Software, Validation. **Naping Xiong:** Data curation, Formal analysis, Methodology, Software, Validation. **Hongwei Shao:** Data curation, Formal analysis, Methodology, Software, Validation. **Zhengming Shan:** Data curation, Formal analysis, Methodology, Software, Validation. **Lizhen Zheng:** Data curation, Methodology, Software, Validation. **Xu Li:** Data curation, Methodology, Software, Validation. **Yanbin Zhu:** Methodology, Supervision, Validation. **Jiankun Xu:** Methodology, Supervision, Validation. **Dick Ho Kiu Chow:** Formal analysis, Methodology, Supervision, Validation. **Ronald Man Yeung Wong:** Conceptualization, Methodology, Project administration, Supervision, Validation, Writing – review & editing. **Ling Qin:** Conceptualization, Funding acquisition, Investigation, Project administration, Resources, Supervision, Visualization, Writing – review & editing. **Wenxue Tong:** Conceptualization, Funding acquisition, Investigation, Project administration, Resources, Supervision, Visualization, Writing – review & editing.

## Declaration of competing interest

The authors declare that they have no competing interests.
