# Supplementary material for: Magnesium-containing intramedullary nails promote fracture healing in type 2 diabetic animal model via recruiting regulatory T cells into the fracture callus
Source: Bioact Mater. 2026 Jul 14;66:573–86. doi: 10.1016/j.bioactmat.2026.07.014 (PMC13383014; doi:10.1016/j.bioactmat.2026.07.014)
Supplement: Multimedia component 1 [file mmc1.docx]

**Supplementary data**

**Supplementary Figure 1**


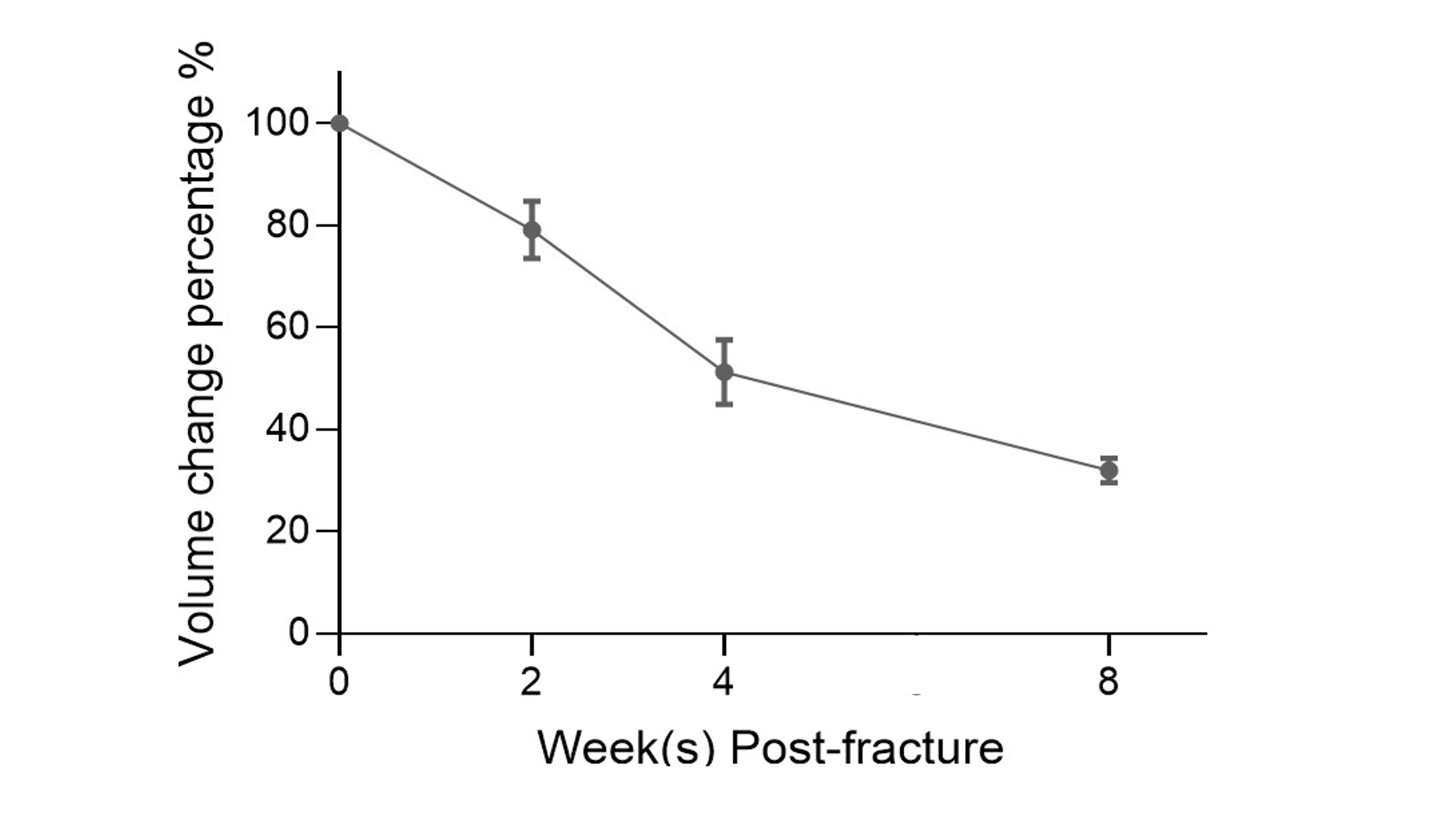


**Supplementary Figure 1. *In vivo* degradation profile of Mg-IMNs in T2D Lepr^db/db^ mice after fracture.** Data are presented as mean ± SD. n = 3

**Supplementary Figure 2**


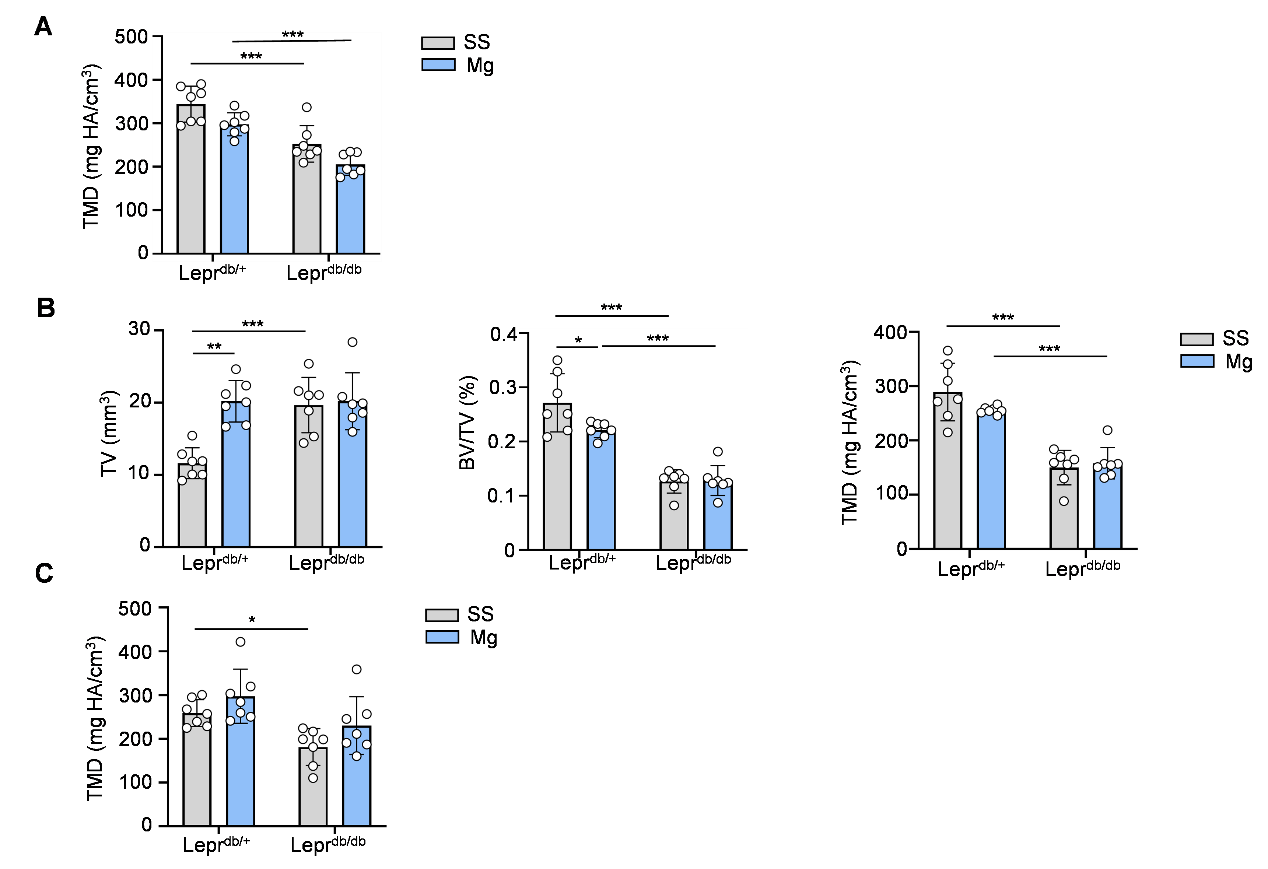


**Supplementary Figure 2. Mg-IMN promotes impaired diabetic fracture healing in Lepr^db/db^ mice.** (A) Quantification of TMD of the callus at 2 weeks post-fracture in Lepr^db/+^+SS, Lepr^db/+^+Mg, Lepr^db/db^+SS, and Lepr^db/db^+Mg groups. ∗∗∗p < 0.001, by two-way ANOVA with Tukey's *post-hoc* test. Data are presented as mean ± SD. n = 7 per group. (B) Quantification of TV, BV/TN, and TMD of the callus at 4 weeks post-fracture in Lepr^db/+^+SS, Lepr^db/+^+Mg, Lepr^db/db^+SS, and Lepr^db/db^+Mg groups. ∗p < 0.05, ∗∗p < 0.01, ∗∗∗p < 0.001, by two-way ANOVA with Tukey's *post-hoc* test. Data are presented as mean ± SD. n = 7 per group. (C) Quantification of TMD of the callus at 8 weeks post-fracture in Lepr^db/+^+SS, Lepr^db/+^+Mg, Lepr^db/db^+SS, and Lepr^db/db^+Mg groups. ∗p < 0.05, by two-way ANOVA with Tukey's *post-hoc* test. Data are presented as mean ± SD. n = 7 per group.

**Supplementary Figure 3**


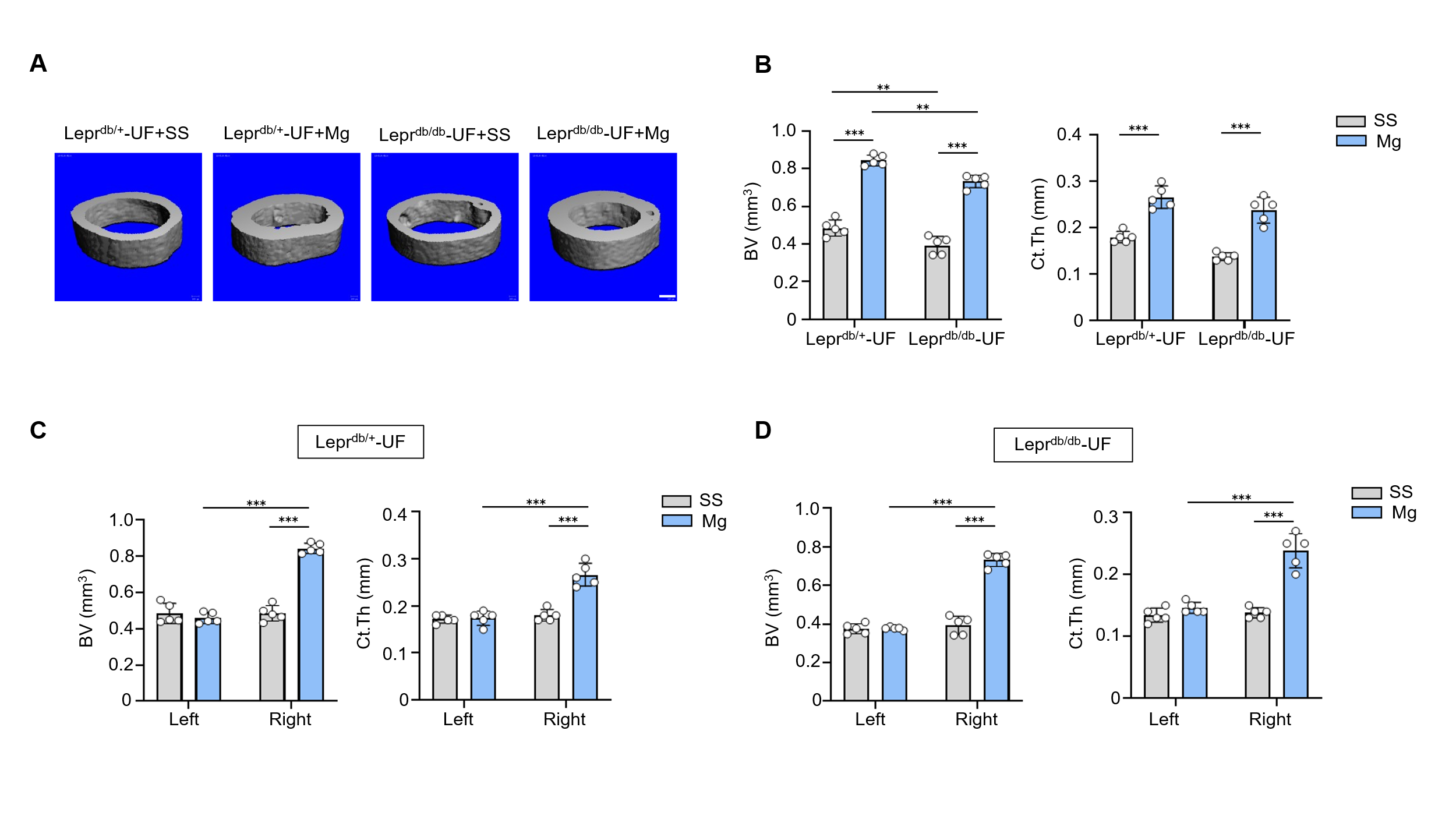


**Supplementary Figure 3. Mg-IMN enhances new cortical bone formation in both** **Lepr^db/+^ and Lepr^db/db^ mice.** Representative micro-CT images (A) and quantification (B) of BV and Ct.Th of the cortical bone from the right femora at 4 weeks post-fracture in Lepr^db/+^-UF +SS, Lepr^db/+^-UF +Mg, Lepr^db/db^-UF +SS, and Lepr^db/db^-UF +Mg groups. Ct.Th: cortical thickness. ∗∗p < 0.01, ∗∗∗p < 0.001, by two-way ANOVA with Tukey's *post-hoc* test. Data are presented as mean ± SD. n = 5 per group. Scale bars, 200 μm. (C) Comparison of BV and Ct.Th of the cortical bone at 4 weeks post-fracture between the left and right femora from Lepr^db/+^-UF +SS and Lepr^db/+^-UF +Mg groups. (D) Comparison of BV and Ct.Th of the cortical bone at 4 weeks post-fracture between the left and right femora from Lepr^db/db^-UF +SS and Lepr^db/db^-UF +Mg groups. ∗∗∗p < 0.001, by two-way ANOVA with Tukey's *post-hoc* test. Data are presented as mean ± SD. n = 5 per group.

**Supplementary Figure 4**

**
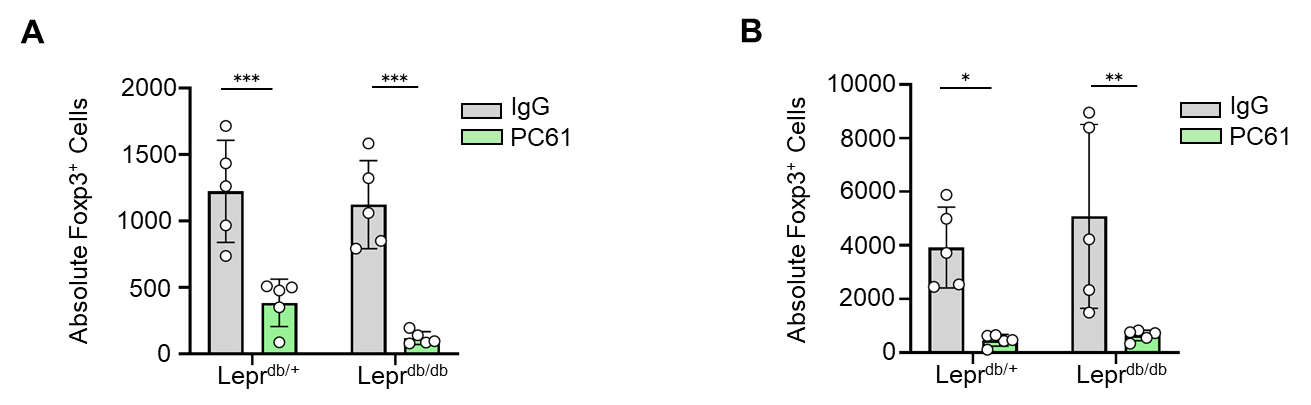
**

**Supplementary Figure 4. PC61 treatment efficiently depletes Tregs.** (A) Absolute Foxp3^+^ cells in blood at 2 weeks post-fracture in Lepr^db/db^SS+IgG, Lepr^db/db^SS+PC61, Lepr^db/db^Mg+IgG, and Lepr^db/db^Mg+PC61 groups. ∗∗∗p < 0.001, by two-way ANOVA with Tukey's *post-hoc* test. Data are presented as mean ± SD. n = 5 per group. (B) Absolute Foxp3^+^ cells in spleen at 2 weeks post-fracture in Lepr^db/db^SS+IgG, Lepr^db/db^SS+PC61, Lepr^db/db^Mg+IgG, and Lepr^db/db^Mg+PC61 groups. ∗p < 0.05, ∗∗p < 0.01, by two-way ANOVA with Tukey's *post-hoc* test. Data are presented as mean ± SD. n = 5 per group.

**Supplementary Figure 5**


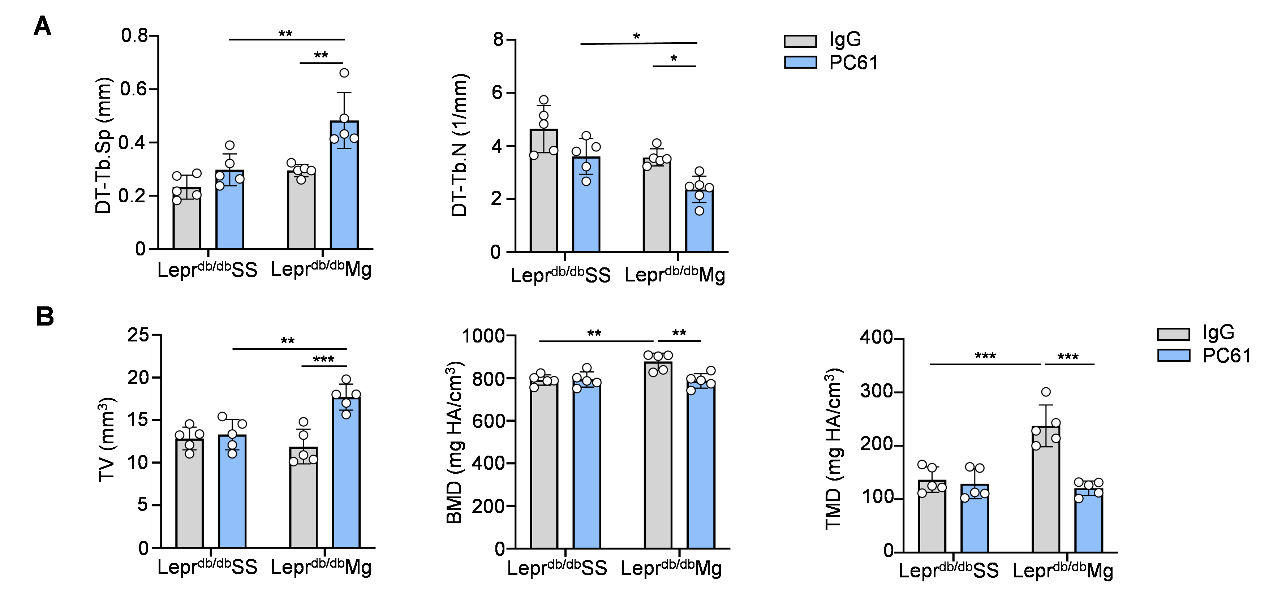


**Supplementary Figure 5. Treg depletion impairs the effect of Mg-IMN on promoting T2D diabetic fracture healing.** (A) Quantification of Tb. Sp and Tb.N of the callus at 2 weeks post-fracture in Lepr^db/db^SS+IgG, Lepr^db/db^SS+PC61, Lepr^db/db^Mg+IgG, and Lepr^db/db^Mg+PC61 groups. ∗p < 0.05, ∗∗p < 0.01, by two-way ANOVA with Tukey's *post-hoc* test. Data are presented as mean ± SD. n = 5 per group. (B) Quantification of TV, BMD, and TMD of the callus at 8 weeks post-fracture in Lepr^db/db^SS+IgG, Lepr^db/db^SS+PC61, Lepr^db/db^Mg+IgG, and Lepr^db/db^Mg+PC61 groups. ∗∗p < 0.01, ∗∗∗p < 0.001, by two-way ANOVA with Tukey's *post-hoc* test. Data are presented as mean ± SD. n = 5 per group.

**Supplementary Figure 6**


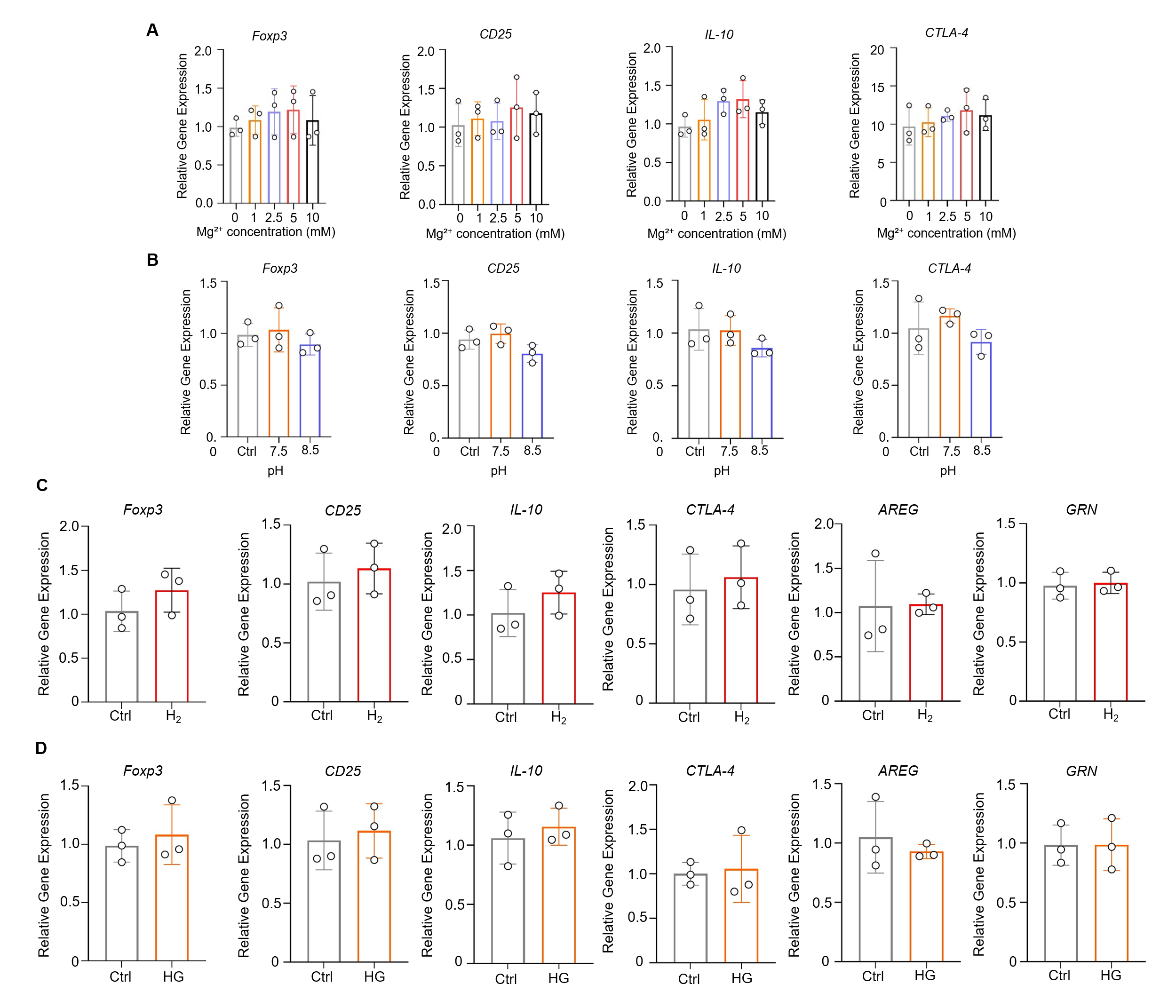


**Supplementary Figure 6. Treg function remains unaffected by Mg-IMN degradation products and high-glucose conditions.** (A) Relative mRNA expression of *FOXP3, CD25, IL-10,* and *CTLA-4* in Tregs under different concentrations of Mg^2+^ treatment. By unpaired two-tailed Student’s *t*-test. Data are presented as mean ± SD. n = 3 per group. (B) Relative mRNA expression of *FOXP3, CD25, IL-10* and *CTLA-4* in Tregs cultured at different pH levels. By unpaired two-tailed Student’s *t*-test. Data are presented as mean ± SD. n = 3 per group. (C) Relative mRNA expression of *FOXP3, CD25, IL-10*, *CTLA-4, AREG,* and *GRN* in Tregs cultured in H_2_-rich medium. By unpaired two-tailed Student’s *t*-test. Data are presented as mean ± SD. n = 3 per group. (D) Relative mRNA expression of *FOXP3, CD25, IL-10*, *CTLA-4, AREG* and *GRN* in Tregs under high glucose conditions. By unpaired two-tailed Student’s *t*-test. Data are presented as mean ± SD. n = 3 per group.

**Supplementary Table 1**

Primers applied in the experimental procedures

| Gene | Forward Primer | Reverse Primer |
| --- | --- | --- |
| *Foxp3* | CCCATCCCCAGGAGTCTTG | ACCATGACTAGGGGCACTGTA |
| *CD25* | AACCATAGTACCCAGTTGTCGG | TCCTAAGCAACGCATATAGACCA |
| *IL-10* | GCTCTTACTGACTGGCATGAG | CGCAGCTCTAGGAGCATGTG |
| *CTLA-4* | TTTTGTAGCCCTGCTCACTCT | CTGAAGGTTGGGTCACCTGTA |
| *Areg* | GGTCTTAGGCTCAGGCCATTA | CGCTTATGGTGGAAACCTCTC |
| *Grn* | ATGTGGGTCCTGATGAGCTG | GCTCGTTATTCTAGGCCATGTG |
